# Endometrial cancer: improving management among increasing incidence rates

**DOI:** 10.1016/j.ebiom.2024.105159

**Published:** 2024-05-13

**Authors:** eBioMedicine

Endometrial cancer is the most common gynaecological cancer in high-income countries. Incidence has been increasing worldwide: between 1990 and 2019, the age-standardised incidence rate increased by 0.69% per year. Although mortality from endometrial cancer has decreased globally, there was a substantial increase in mortality in more than 40% of countries during that same timeframe, as reported in *Gynecologic Oncology*. Early diagnosis is associated with favourable outcomes, but for women who are diagnosed at a later stage or who have recurrent or metastatic disease, prognosis remains poor.

Most cases of endometrial cancer are diagnosed during perimenopause or post-menopause after presentation with atypical vaginal bleeding. Pelvic ultrasound scan is the most common follow-up procedure but has a low specificity for endometrial cancer, meaning that a painful, invasive endometrial biopsy might be required. As fewer than 10% of people who have symptoms have an underlying malignancy, there is a clear need to develop minimally invasive screening tools. In April, 2024, a study published in *eBioMedicine* evaluated the diagnostic use of proteomic signatures derived from plasma and cervicovaginal fluid collected from 118 women who were symptomatic and post-menopausal. They found that the protein signature derived from the cervicovaginal fluid more accurately detected early-stage cancers, advanced-stage cancers, and aggressive phenotypes compared with the plasma-derived signature (AUC 0.95 [95% CI 0.91–0.98] *vs* 0.87 [0.81–0.93]). The authors proposed a five-biomarker panel combining HPT, LG3BP, FGA, LY6D, and IGHM, which should be evaluated in larger and more diverse cohort studies.

The use of cervicovaginal sampling to facilitate molecular subtyping of endometrial cancer has also been explored. A prospective case–control study published in *eBioMedicine* in July, 2023, investigated the use of cervicovaginal samples to detect genetic alterations and enable molecular classification of tumours. The authors designed a custom panel to sequence 47 genes, derived from previous work published in *Cancer Epidemiology*. Overall, the test identified 73% of endometrial cancer cases. They were able to classify people with endometrial cancer into four prognostically relevant molecular subtypes, defined by the Cancer Genome Atlas. In clinician-collected samples, the custom panel outperformed cervical cytology (AUC 0.81 [95% CI 0.75–0.87] *vs* 0.67 [0.60–0.75]). Importantly, a validation group of self-collected samples from 111 women was also included, showing similar performance to clinician-collected swabs (0.85 [0.75–0.95]). Self-collection has previously been shown to improve screening uptake for other gynaecological conditions, such as testing for human papillomavirus (reviewed in *BMC Women's Health*). As highlighted in *The Lancet Oncology* in March, 2023, the molecular classification of endometrial cancer improves patient outcomes by enabling targeted treatments.

Patients diagnosed with low-stage endometrial cancer (eg, 1A and 1B) typically have a favourable prognosis with surgical treatment only, but 7% of such tumours recur. For these patients, adjuvant therapy concurrent with surgery would be beneficial, but there is currently no marker to robustly identify endometrioid tumours with a high recurrence risk. In a single-cell profiling study published in *eBioMedicine* in June, 2023, the authors characterised the cellular composition of endometrial tumours to improve understanding of how they spread. By comparing tissues from 17 recurrent tumours and 19 non-recurrent tumours, the authors found that the distribution of major cell populations could not explain the difference in recurrence of tumours. Further analysis revealed higher expression of vimentin in epithelial cells from non-recurrent tumours than from recurrent tumours and an association between low vimentin expression and poor recurrence-free survival. Further follow-up of pre-operative samples from 518 patients found that loss of vimentin expression or low vimentin expression were significantly associated with poor recurrence-free survival.

An increasing number of people of reproductive age are being diagnosed with endometrial cancer. In the USA, 11.6% of diagnoses were in patients younger than 50 years according to a retrospective analysis of patients in the US National Cancer Database from 2005 to 20 published in *Cancers* in March, 2024. The increasing incidence in this age group is thought to be due to changing risk factors, such as the increasing prevalence of obesity and delayed childbirth. For individuals of reproductive age who want to retain their childbearing ability, preserving fertility is a crucial consideration in the management of endometrial cancer. Fertility-sparing hormonal treatments, such as progestogen, are an option but are not recommended for some subtypes of endometrial cancer. To examine the underlying biology of early-onset endometrial cancer, the authors of an April, 2024, *Nature Genetics* paper conducted a multi-omics study of 215 patients, revealing different molecular characteristics from late-onset endometrial cancer. Endometrial cancer that was insensitive to progestogen was associated with higher BMI and *SIGLEC10*^*Q144K*^ mutations; *SIGLEC10*^*Q144K*^ mutations are hypothesised to lead to increased SIGLEC-10 expression and downstream activation of oestrogen signalling. Intriguingly, a molecular signature associated with the exposome was observed, indicating the possibility that the increase in incidence could also be related to changes in environmental exposures.

Genetics also influence endometrial-cancer risk. A genome-wide association study of 4951 women who were post-menopausal published in *eBioMedicine* in February, 2024, identified four single-nucleotide polymorphisms involved in the regulation of oestrone concentration that were associated with endometrial cancer risk. In women who are post-menopausal, oestrone is one of the main circulating oestrogens. This association, which was replicated in a dataset from the UK Biobank, was independent of other risk factors (eg, BMI, parity, and diabetes). A review summarising the progress and insights from genome-wide association studies of people with endometrial cancer in the 10 years since the first study was conducted was published in *eBioMedicine* in March, 2022. Because of the progress in understanding genetics contributing to endometrial-cancer risk, polygenic risk scores, when integrated with other known risk factors, will hopefully assist screening within the next decade.

Endometrial-cancer mortality is predicted to continue to increase in the coming years, one of only a few types of cancer for which this is predicted. This prediction, and the increasing incidence of endometrial cancer worldwide, emphasise the urgency for continued research into accurate diagnostic methods that can ultimately facilitate personalised management and treatment. *eBioMedicine* encourages studies addressing this crucial area to combat the burden of endometrial cancer.

